# Comparison of postoperative pulmonary complications and intraoperative safety in thoracoscopic surgery under non-intubated versus intubated anesthesia: a randomized, controlled, double-blind non-inferiority trial

**DOI:** 10.1007/s13304-024-01935-y

**Published:** 2024-08-10

**Authors:** Lingfei Wang, Dan Wang, Yanmei Zhang

**Affiliations:** Department of Anesthesiology, Liaoning Cancer Hospital & Institute, Shenyang, 110042 Liaoning China

**Keywords:** Non-intubated anesthesia, Spontaneous breathing, VATS, Post-operative pulmonary complications

## Abstract

**Purpose:**

Traditional anesthesia for video-assisted thoracoscopy (VATS) such as double-lumen tracheal intubation (DLT) and one-lung ventilation (OLV), may lead to post-operative pulmonary complications (PPCs). Non-intubation VATS (NIVATS) is an anesthetic technique that avoided DLT and OLV, maybe avoiding the PPCs. So we hypothesized that NIVATS would non-inferiority to intubation VATS (IVATS) in the risk of developing PPCs and some safety indicators.

**Methods:**

This study is a randomised, controlled, double-blind, non-inferiority trial, 120 patients were randomly assigned to the NIVATS group and IVATS group according to 1:1. The primary outcome was the incidence of PPCs with a pre-defined non-inferiority margin of 10%. The second outcome was the safety indicators, including the incidence of cough/body movement, hypoxemia, malignant arrhythmia, regurgitation and aspiration, and transferring to endobronchial intubation intraoperatively (The malignant arrhythmia was defined as an arrhythmia that caused hemodynamic disturbances in a short period of time, resulting in persistent hypotension or even cardiac arrest in the patient).

**Results:**

There was no significant difference in demographic indicators such as gender and age between the two groups. The incidence of PPCs in the NIVATS group was non-inferior to that in the IVATS group (1.67% vs. 3.33%, absolute difference: − 1.67%; 95%CI − 7.25 to 3.91). In additionan, no significant differences were found between the two groups for the incidence of cough/body movement (10.00% vs. 11.67%, *p* = 0.77), the incidence of hypoxemia (25% vs. 18.33%, *p* = 0.38), the incidence of malignant arrhythmia (1.67% vs. 6.67%, *p* = 0.36), the incidence of regurgitation and aspiration (0% vs. 0%, *p* > 0.999) and the incidence of transferring to endobronchial intubation intraoperatively (0% vs. 0%, *p* > 0.999).

**Conclusion:**

We conclude that when using the non-intubation anesthesia for VATS, the incidence of PPCs was not inferior to intubation anesthesia. Furthermore, NIVATS had little effect on perioperative safety.

## Introduction

In recent years, the swift advancement of thoracic surgery has been predicated on the Double-lumen bronchial tube (DLT), which was a milestone event in the development history of thoracic surgery and anesthesia [1]. The ability of DLT to deliver one-lung ventilation (OLV) and a favorable surgical field has accelerated the process of video-assisted thoracic surgery (VATS), leading to precise operations with minimal invasion [2].

Nevertheless, extensive research [3–5] conducted in the past few years has revealed that the intubated VATS (IVATS) is associated with numerous complications and side injuries. The strong stimulation of double-lumen tube intubation leads to dramatic fluctuation of intraoperative circulation and postoperative sore throat in patients. The residual muscle relaxants cause prolonged muscle recovery time, insufficient respiratory muscle strength, and decreased effective ventilation, further leading to postoperative pulmonary atelectasis and hypoxemia. Meanwhile, mechanical ventilation for OLV can also result in adverse reactions such as mechanical ventilation-associated lung injury, ventilator-associated pneumonia, and re-expansion lung injury. Furthermore, it has been shown [6] that postoperative pulmonary complications (PPCs) are common after IVATS, which not only causes pain to patients, but also seriously affects postoperative recovery, increases hospitalization costs, and prolongs the stay of hospitalization [7]. In thoracic surgical patients, the incidence of PPCs (14–59%) is higher than in other types of major surgery [8–10].

In order to reduce intraoperative and postoperative complications caused by DLT, and minimize the impact of OLV as much as possible, non-intubated VATS (NIVATS) has gradually been applied in thoracic surgery [11]. NIVATS is a surgical technique that employs a non-invasive airway device to administer general anesthesia with the assistance of local and/or regional block anesthesia techniques to maintain the patient's spontaneous breathing during VATS [12]. NIVATS is an emerging anesthesia that avoids double-lumen tracheal intubation, OLV and mechanical ventilation [13]. Although previous studies [14–16] have demonstrated the feasibility and safety of NIVATS, it still suffers from complications arising from the regional and local anaesthesia technique itself as well as respiratory, haemodynamic and neurological events, and in NIVATS with open pneumothorax, ventilation of the collapsed lungs can be compromised, leading to some degree of hypoxaemia, hypercapnia and acidosis. In addition, the effect of NIVATS on the incidence of PPCs has not been clarified. Therefore, this study used a randomized, controlled and double-blind method to investigate whether the incidence of PPCs in the NIVATS group was not inferior to that in the IVATS group for patients with BMI < 25 kg/m^2^ and ASA I–II.

## Methods

### Study design and ethics

This was a randomized, parallel-controlled, double-blind, and non-inferiority clinical trial at the Department of Anesthesiology of our hospital between September 2020 to October 2021. The study protocol was approved by our hospital ethics committees (2020[05] No.20200459). Written informed consent was obtained from all participants or their legal guardians. The trial was registered with the Chinese Clinical Trial Registry (http://www.chictr.org.cn, No.ChiCTR2000038041).

### Patients

**Fig. 1:**
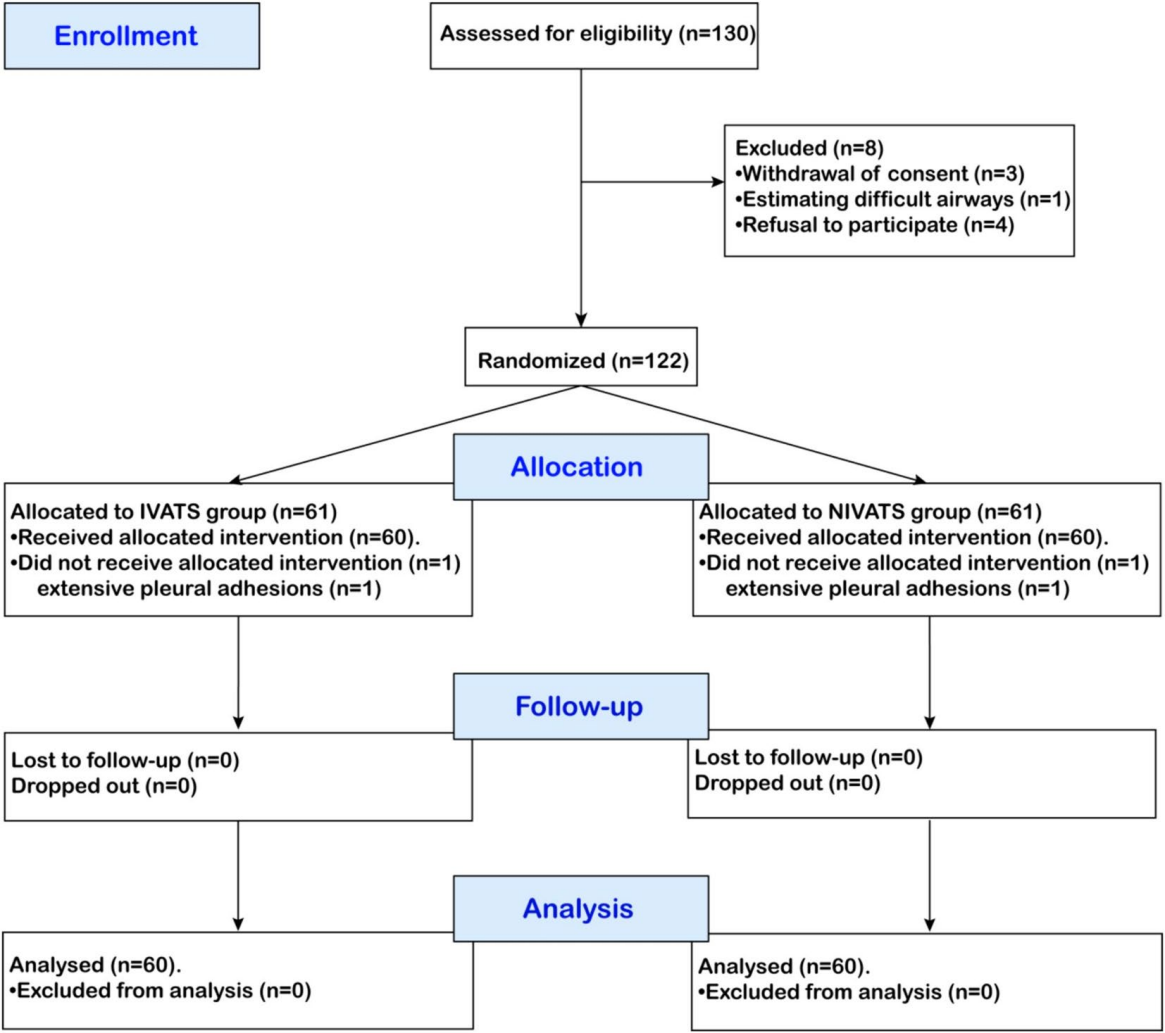
CONSORT diagram describing patient progress through each stage of the randomized trial

A total of 130 patients were enrolled in the trial based on the specified inclusion criteria. Among them, 10 patients were either excluded or dropped out, and eventually, 120 patients completed the trial. (Fig. 1).

We recruited patients who met the following inclusion criteria: (1) The American Society of Anesthesiologists (ASA) [17] grade I–II. (2) No history of thoracic disease. (3) No history of severe cardiovascular disease., including severe hypertension (BP ≥ 180/100 mmHg), frequent atrial or ventricular premature beats (≥ 5 bpm/min), congenital valvular lesions, heart failure (EF ≤ 50%), coronary heart disease (unstable angina pectoris, myocardial infarction), cerebral infarction, etc. (4) Body mass index (BMI) < 25 kg/m^2^. (5) Mallampati grade [18]: I–II. The exclusion criteria: (1) The patients who stopped using anticoagulation therapy and/or antiplatelet therapy less than a week preoperatively. (2) Abnormal airway or predictable difficult airway (Tracheostomy, history of pharyngeal surgery, foreseeable difficulties in mask ventilation or difficult intubation). (3) Bronchiectasis and asthma, or any respiratory disorder leading to decreased lung function. (4) Diameter of tumor > 5 cm, centrally located or local metastasis or complex surgery. (5) Predictable and unpredictable massive haemorrhage or change in surgical method. (6) Epilepsy or any other nervous system disease. (7) Patients who need to switch to tracheal intubation or withdraw midway intraoperativly.

### Randomization and blinding

Patients were randomized into either the NIVATS or IVATS groups at a ratio of 1:1 using a computer-generated random sequence and a sealed envelope method administered by a medical statistician. The anesthetist opens the envelope after the patient enters the operating room, retrieves the grouping information, and administers the medication accordingly. The anaesthetist was responsible only for intraoperative anaesthesia management and the thoracic surgeons were responsible only for surgical performance, and neither participate in the study's design, data recording, or analysis. Except for the anesthesiologist and thoracic surgeons, all study personnel, and patients remained unaware of the group assignments. The results of the grouping were unveiled only after the completion of data analysis.

### Sample size calculation

We computed a sample size of 60 patients using the "sample size" package in R, a two-sided significance level and a detection rate was set at 0.05 and 0.8 respectively, with a pre-defined non-inferiority margin of 10%. According to the clinical judgment and the available data at the time of trial design, 10% of the non-inferiority margin was set as the clinically relevant difference of PPCs in previous studies [19, 20]. After considering potential withdrawal (30%) and the increase in sample size for non-parametric analysis (15%), 120 patients (60 in each group) were calculated as the final sample size. Also, to verify the reliability of the sample size of this study, statistical power analysis (Cohen’s *d* = − 0.33882, 1–*β* = 0.957) was conducted and proved that the sample size selected for this study (*n* = 120) was sufficient to generate statistical power.

### Study procedure

Both groups of patients underwent single-port VATS, with the surgical type being lung cancer radical resection or lung wedge resection. All anesthesia was performed by the same senior anesthesiologist (more than ten years as an anesthesiologist for thoracic anesthesia), and all operations were completed by the same group of surgeons who experienced the surgical procedures. Penehyclidine hydrochloride (0.5 mg) was given as a premedicant. The patients were admitted to the operating theatre in a lying position, and venous access to the upper limbs was established. Continuous monitoring included electrocardiogram (ECG), heart rate (HR), pulse oximetry (SpO_2_), non-invasive cuff blood pressure (NIBP), and bispectral index (BIS) and brain oxygen saturation (rSO_2_). Before anesthesia induction, radial artery puncture cannulation on the non-operative side was performed under local anesthesia for continuous monitoring of invasive arterial pressure and arterial blood gas (ABG) analysis intermittently.

Anesthesia induction using dexmedetomidine hydrochloride (0.5–1 μg kg^−1^ h^−1^), followed by propofol (plasma target concentration: 1–2.5 μg ml^−1^) and remifentanil (plasma target concentration: 0.5–2 ng ml^−1^) by target-controlled infusion (TCI). When bispectral index (BIS) value reached 50 ± 10 [21], the laryngeal mask airway (LMA) was inserted in the NIVATS group, DLT was inserted in IVATS group after rocuronium injection (0.7–0.9 mg kg^−1^) and located by fiberoptic bronchoscopey. Patients in the NIVATS group were kept breathing spontaneously with oxygen support though LMA, while those in the IVATS group were ventilated with positive pressure. If intraoperative spontaneous breathing inconveniences, the anaesthetist will regulate the patient's tidal volume and respiratory rate by adjusting the rate of anaesthetic drug infusion, but not intermittent positive pressure ventilation. The infusion of propofol and remifentanil during operation was mainly adjusted according to BIS value (maintained 40–50), respiratory and circulation indexes.

The parathoracic nerve block (PVB) was achieved with 20 ml of 0.25% ropivacaine (10 ml each segment, AstraZeneca) to T4–T5 segments in both group. In the NIVATS group, the anesthesia was maintained by delivering 100% oxygen at a rate of 4 L/min. 2% lidocaine 3–5 ml for vagal nerve block and lung spraying with 15–20 ml of 0.5% ropivacaine, repeat the administration of the above processing every 2 h [22]. Dexmedetomidine was stopped at the beginning of the skin suture, propofol and remifentanil were stopped 5 min before the skin suture completion. When hypotension (mean arterial pressure, MAP) less than 80% of basal MAP for more than 1 min), the depth of anesthesia was adjusted or norepinephrine was administered at 0.05 ~ 0.10 μg kg^−1^ h^−1^ until MAP exceeded 80% of basal. Conversely, in the event of hypertension (MAP exceeding 120% of basal MAP for more than 1 min), the depth of anesthesia was adjusted or urapidil 10 mg was administered. Bradycardia (HR < 60 beats/min) was treated with atropine.

The patients were subsequently transferred to the Post-Anesthesia Care Unit (PACU). The tracheal tube was removed once the patient could open their eyes, breathe spontaneously, have adequate tidal volume, and achieve circulatory stability. When the patient's muscle strength was not adequately restored, neostigmine was administered; propofol was used to treat intolerance to mechanical ventilation; and fentanyl was administered to address pain experienced after awakening.

### Outcome measures

The primary outcome was the incidence of PPCs. Diagnosis is confirmed when four or more criteria are present on a postoperative day, as follows: (1) New abnormal breath sounds on auscultation different from in the preoperative assessment. (2) Production of yellow or green sputum different from in the preoperative assessment. (3) SpO_2_ < 90% on room air on more than one consecutive postoperative day. (4) Maximum oral temperature > 38 ℃ on more than one consecutive postoperative day. (5) Chest radiography report of collapse or consolidation. (6) An unexplained white cell count greater than 11 $\times$ 10^9^/L. (7) Presence of infection on sputum culture report. (8) Physician's diagnosis of pneumonia, lower or upper respiratory tract infection, an undefined chest infection, or prescription of an antibiotic for a respiratory infection.

The secondary outcomes included the incidence of the intraoperative safety indicators. Cough/body movements (Cough/body movement was assessed by the surgeon: the presence of an airway response or any movement of the trunk or extremities by the patient in response to surgical stimuli was classified as cough/body movement), hypoxemia (SPO_2_ < 90%), regurgitation and aspiration (Doctors found stomach contents in the patient's upper airway or trachea), the incidence of transfer to tracheal intubation, and malignant arrhythmia (Malignant arrhythmia was defined as an arrhythmia that caused hemodynamic disturbances in a short period of time, resulting in persistent hypotension or even cardiac arrest in the patient).

In addition, this study also recorded intraoperative medication use, various vital signs at each time point, and postoperative recovery quality. Postoperative recovery quality included duration of awakening, extubation (LMA) time, chill, agitation, nausea/vomiting, sore throat, fasting time, exhausting time, retention time of thoracic catheter, et al. Various vital signs such as rSO_2_, ABG, and BIS were recorded at the following time points, T0: before anesthesia. T1: 5 min after intubation. T2: 30 min after surgery begins (OLV). T3: 60 min after surgery begins (OLV). T4: 90 min after surgery begins (OLV). T5: immediately after the operation (lung recruitment). T6: 15 min after extubation (LMA). ABG was performed through radial artery catheterization at T0, T2, T3, T4, and T6 time points, including PaCO_2_, PH, HCO_3_^−^, and BE. The visual analog scale (VAS) was from 0 to 10, the VAS and the dosage of the analgesia pump were also evaluated at 2 h, 6 h, 12 h, 24 h, and 48 h postoperatively. In addition, demographic indicators such as gender, age, BMI index, surgical site, etc. were also collected in detail for both groups.

### Safety of intervention

To ensure the safety of patients in the NIVATS group, we have established safety assessment standards [23]: 1. Continuous SPO_2_ < 90%, PaCO_2_ > 80 mmHg did not improve 5 min after adjusting. 2. Cough reflex that cannot be inhibited by spraying the lung surface and vagus nerve block. 3. Intraoperative reflux or misaspiration. 4. Intraoperative hemodynamic cannot be maintained. If the above situation occurs, it will be converted to endotracheal intubation. The steps for intubation: 1. Insert single-lumen tracheal intubation through a visual laryngoscope and then insert a bronchial blocker. 2. If the anesthesiologist believes that inserting DLT is not difficult, they can directly intubate the DLT.

### Statistical analysis

PPCs are compared between NIVAS and IVAS in this non-inferiority trial. Prior research has established a 10% minimum clinically significant difference for PPCs; therefore, the current study established a 10 margin of non-inferiority for the difference in PPCs between groups. 95% confidence interval (CI) around the risk difference and *P* values for non-inferiority was calculated using R statistical software (version 4.2.1; R Core Team) and SAS statistical software (version 9.4; SAS Institute Inc., Cary, NC, USA).

All statistical analyses were performed using IBM SPSS 23.0. The primary and secondary outcomes analyses were done in the per-protocol set, consisting of eligible, randomised patients with no major protocol deviations affecting treatment efficacy. Quantitative data was tested by Kolmogorov–Smirnov. The normally distributed quantitative data were described as mean ± standard deviation ($\overline{x }\pm s$), and independent samples t-tests were employed for group comparisons. Non-normally distributed data were presented as medians and quartiles [M(P25, P75)], and the comparison between groups was performed by the Mann–Whitney *U* test. Qualitative data are described as percentages, and comparisons between groups are made by chi-square tests. Comparisons of indicators at each observation point were analyzed using repeated-measures analysis of variance (ANOVA). For Non-normally distributed data, a generalized linear model was employed for analysis. All statistical tests were 2-sided and a value of *P* < 0.05 was considered to be statistically significant.

## Result

### Patients’ disposition and baseline characteristics

A total of 130 patients were enrolled from the surgical planning list, four patients declined to participate, one patient withdrew for unspecified reasons prior randomization, and three were excluded for difficult airway assessment prior randomization. 122 patients were randomly allocated into two groups, after randomization, two patients with extensive pleural adhesions were excluded, thus, 120 participated in the final analysis (Fig. 1). The baseline characteristics such as gender, age, ASA classification, BMI, EF%, history of operation, education, surgical method, surgical site and intraoperative data were well-balanced between the two groups, except for urine volume (Table 1).

**Table 1:** Demographic characteristics and intraoperative data

	IVATS (*n* = 60)	NIVATS(*n* = 60)	*P*-value
Age, (year)	51.43 ± 7.65	51.92 ± 6.39	0.71
Gender, *n* (%)			0.25
Male	24 (40)	18 (30)	
Female	36 (60)	42 (70)	
Height, (cm)	165.38 ± 7.65	163.72 ± 5.48	0.14
Weight, (kg)	63.35 ± 7.93	60.9 ± 7.69	0.09
BMI, (kg/m^2^)	21.15 ± 1.60	22.59 ± 1.60	0.06
EF%	61.47 ± 5.24	61.43 ± 4.5	0.97
ASA Ӏ, *n* (%)	24 (40)	29 (48.33)	0.36
Diabetes, *n* (%)	9 (15)	9 (15)	> 0.999
Cardiovascular disease, *n* (%)	2 (3.33)	1 (1.67)	> 0.999
Hypertension, *n* (%)	15 (25)	10(16)	0.26
History of operation, *n* (%)	11(18.33)	12(20.00)	0.82
Education, *n* (%) (High school or above)	31 (51.67)	25 (41.67)	0.27
Surgical method, *n* (%)			0.47
Lobectomy	51 (85)	48 (80)	
Wedge	9 (15)	12 (20)	
Surgical site (Left), *n* (%)	9 (15)	9 (15)	> 0.999
Anesthesia duration, (min)	168.55 ± 40.29	180.03 ± 38.45	0.11
Operation duration, (min)	148.08 ± 36.72	145.88 ± 40.41	0.58
Blood loss, (> 100 ml)	53 (88.33)	46 (76.67)	0.09
RBC infusion, (U)	0 (0)	0 (0)	> 0.999
Colloid volume, (ml)	238.25 ± 27.75	247.67 ± 49.62	0.20
Crystalloid volume, (ml)	741 ± 57.10	751.17 ± 91.82	0.59
Urine volume, (ml)	279.67 ± 108.52	247.67 ± 49.62	< 0.01
Lymph node dissection, (pcs)	10.97 ± 1.76	10.43 ± 2.16	0.14

Data are presented as the mean ± SD, median (interquartile range), or number of patients (%)

*BMI* Body Mass Index, *ASA* American Society of Anesthesiologists, *EF* Ejection fraction, *SD* standard deviation;

### Primary outcome

**Fig. 2:**
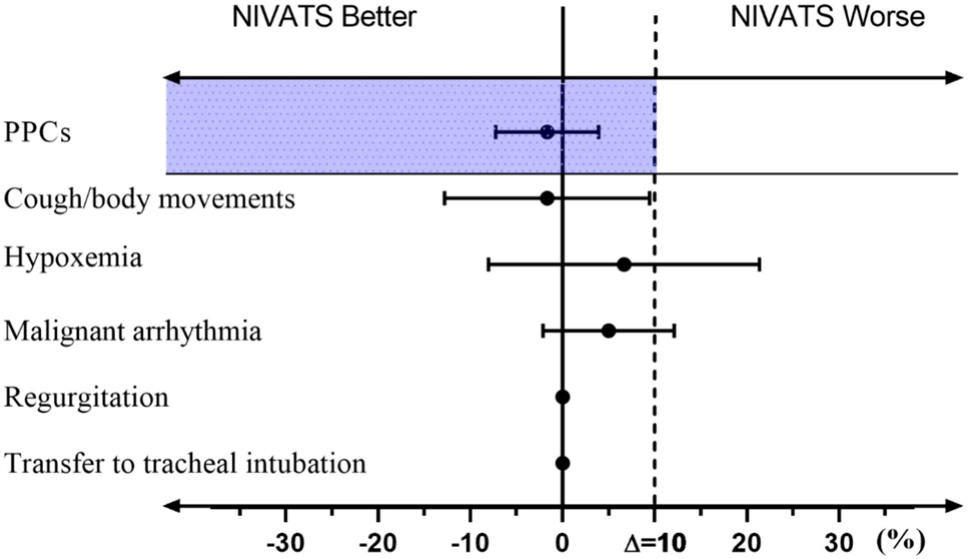
Group differences in the incidence of PPCs and intraoperative safety indicators (mean 95% CI). The 10% non-inferiority margin is only used for analysis of the primary outcome (PPC_S_)

The incidence of PPCs in the NIVATS group was non-inferior to that in the IVATS group (1.67% vs. 3.3%; absolute difference: − 1.67%; 95%CI − 7.25 to 3.91%) (Table 2 and Fig. 2).

**Table 2:** The Incidence of PPC

	IVATS (*n *= 60)	NIVATS (*n* = 60)	Difference (95% CI)	Non-inferiority test *P* value
PPCS	2 (3.33%)	1 (1.67%)	− 1.67% (− 7.25, 3.91)	> 0.999

Data are presented number of patients (%)

*PPCs* post-operative pulmonary complications, *CI* confidence interval

### Secondary outcomes

The intraoperative adverse event of the study is presented in Table 3. There were no significant differences were found between the two groups for the incidence of cough/body movement (10.00% vs. 11.67%, *P* = 0.77), the incidence of hypoxemia (25% vs. 18.33%, *P* = 0.38), and the incidence of transferring to endobronchial intubation intraoperatively (0% vs. 0%, *P* > 0.999). There was no statistical difference in other adverse event such as regurgitation and aspiration [0(0%) vs. 0(0%); *P* > 0.999] and malignant arrhythmia [4(6.67%) vs. 1(1.67%); *P* = 0.36] between the two groups (Table 3 and Fig. 2).

**Table 3:** The intraoperative safety indicators

	IVATS (*n* = 60)	NIVATS (*n* = 60)	*P-*value
Cough/body movement	7 (11.67)	6 (10.00)	0.77
Hypoxemia	11 (18.3)	15 (25)	0.38
Transferred to tracheal intubation	0	0	> 0.999
Regurgitation and aspiration	0	0	> 0.999
Malignant arrhythmia	1 (1.67)	4 (6.67)	0.36

Data are presented number of patients (%)

The perioperative medicine application between the two groups is presented in Table 4. The dosage of sufentanil, propofol, remifentanil, and dexmedetomidine in the NIVATS group is significantly lower than that in the IVATS group (*P* < 0.05). In addition, the dosage of vasoactive drugs such as norepinephrine in the NIVATS group was significantly lower than that in the IVATS group (*P* < 0.05). Furthermore, compared with the IVATS group, the dosage of drugs in PACU such as fentanyl_PACU,_ propofol_PACU,_ and neostigmine_PACU_ in the NIVATS group was significantly reduced (*P* < 0.05) (Table 4).

**Table 4:** The comparison of medicine application in perioperative

	IVATS (*n* = 60)	NIVATS (*n* = 60)	*P-*value
Sufentanil, (μg)	40.38 ± 5.95	12.28 ± 4.48	< 0.0001
Propofol, (mg)	843.39 ± 320.06	729.91 ± 215.37	0.03
Remifentanil, (μg)	654.98 ± 306.78	510.09 ± 275.56	< 0.01
Dexmedetomidine, (μg)	93.04 ± 23.18	117.75 ± 26.32	< 0.0001
Norepinephrine, (μg)	490.03 ± 308.32	328.9 ± 254.23	< 0.01
Atropine, *n* (%)	7 (11.67)	5 (8.33)	0.54
Fentanyl _PACU_, *n* (%)	13 (21.67)	5(8.33)	0.04
Propofol _PACU,_ *n* (%)	10 (16.67)	2 (3.33)	0.03
Neostigmine _PACU,_ *n* (%)	7(11.67)	0 (0)	0.01
Uradil _PACU,_ *n* (%)	9 (15.00)	9 (15.00)	> 0.999

Data are presented as the mean ± SD or number of patients (%)

**Fig. 3:**
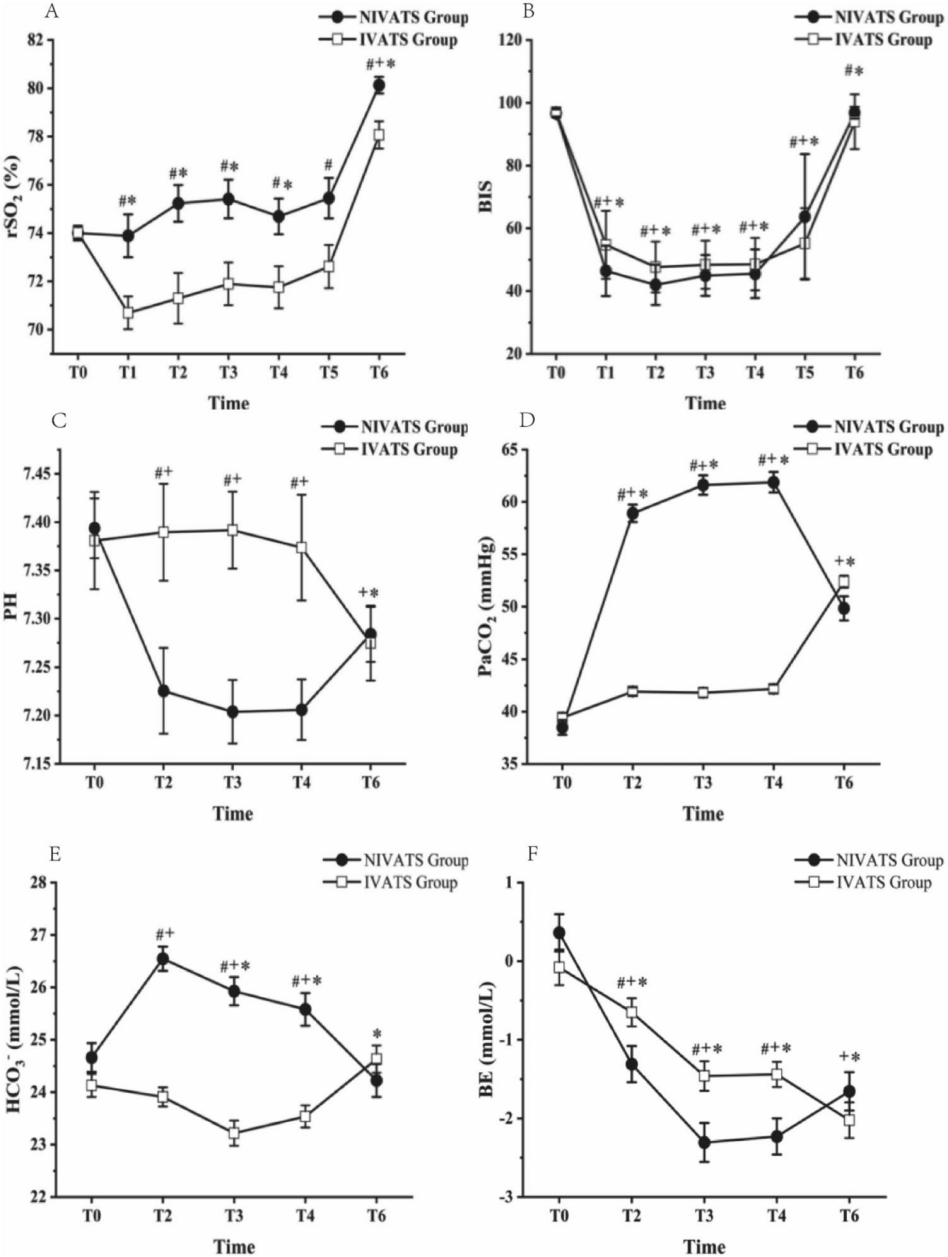
The changes in the vital signs from T0 to T6. Perioperative vital signs include **A** rSO2, **B** BIS, **C** PH, **D** PaCO2, **E** HCO3-, **D** BE. T0: before anesthesia. T1: 5 min after intubation. T2: 30 min after surgery begins (OLV). T3: 60 min after surgery begins (OLV). T4: 90 min after surgery begins (OLV). T5: immediately after the operation (lung recruitment). T6: 15 min after extubation (LMA). #*P* < 0.05: NIVATS vs IVATS; + *P* < 0.05: vs T0 time point in NIVATS group; *P < 0.05: vs T0 time point in IVATS group

The changes of the rSO_2_, BIS, and ABG results from T0 to T6 are presented in Fig. 3. After anesthesia induction, rSO_2_ in the NIVATS group was significantly higher than that in the IVATS group at time points T1 to T6 (*P* < 0.05) (Fig. 3A). BIS in NIVATS group at T1 to T4 was lower than that in IVATS group, but higher at immediately at T5 and T6 (Fig. 3B). In the matter of ABG, compared with IVATS group, the PH, PaCO_2_ and BE in NIVATS group was significantly elevation intraoperatively (T2 to T4) (*P* < 0.05) (Fig. 3C, D, F), and the HCO^3−^ was significantly increased at T2 to T4, T6 in NIVATS group (*P* < 0.05) (Fig. 3E).

**Fig. 4:**
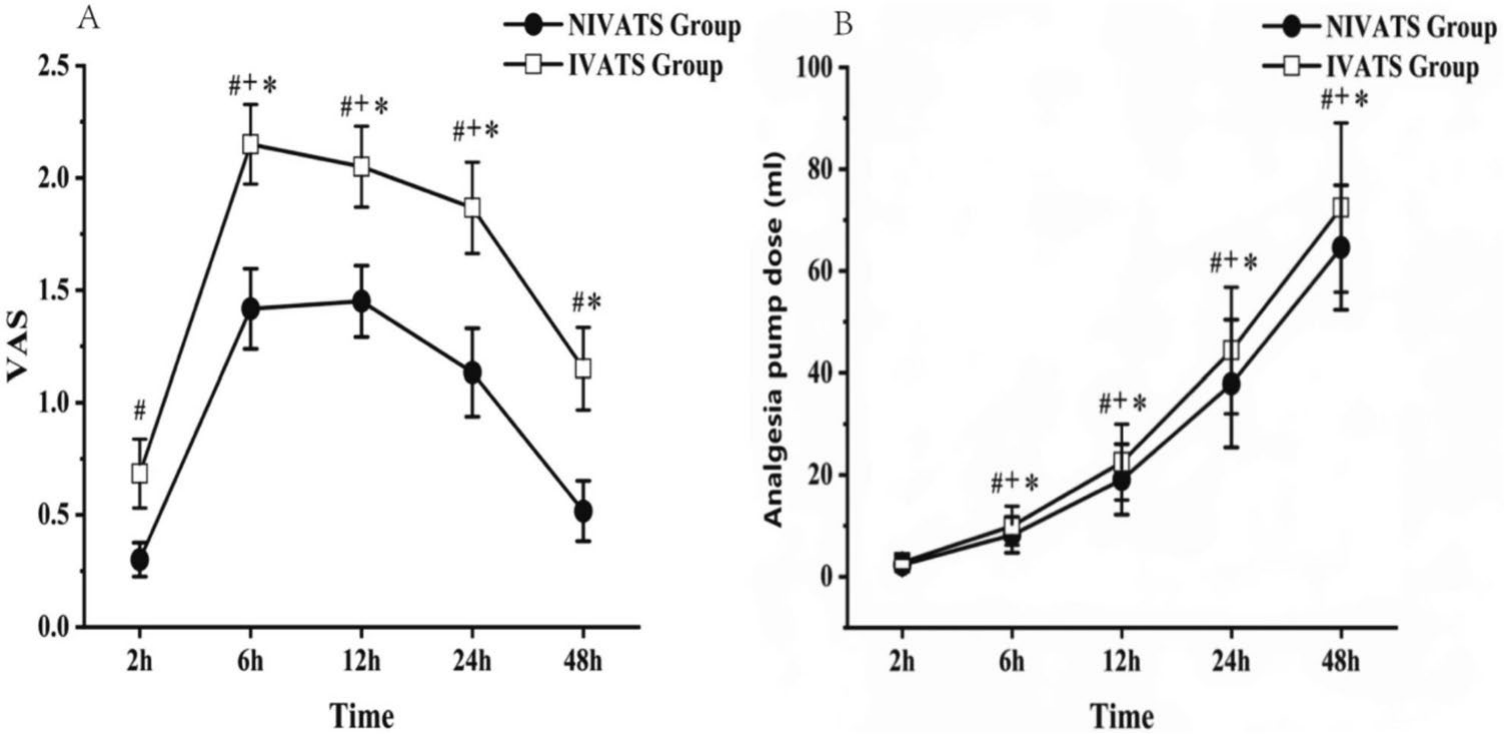
The VAS and the dosage of the analgesia pump at 2 h, 6 h, 12 h, 24 h, and 48 h postoperatively. #*P *< 0.05: NIVATS vs IVATS; + *P* < 0.05: vs 2 h in NIVATS group; *P < 0.05: vs 2 h in IVATS group

The VAS and the dosage of the analgesia pump are shown in Fig. 4. The VAS score was significantly lower in the NIVATS group than in the IVATS group at 2 h, 6 h, 12 h, 24 h, and 48 h postoperatively (*P* < 0.05) (Fig. 4A), and the dose of analgesic was also significantly less than in the IVATS group at 6 h, 12 h, 24 h, and 48 h postoperatively (*P* < 0.05) (Fig. 4B).

The results of recovery indicators between the two groups are presented in Table 5. The duration of awakening, duration of extubation, VAS extubation _5 min_, VAS extubation _15 min_, and the incidence of chill, agitation, and sore throat in the NIVATS group were significantly lower than those in the IVATS group (*P* < 0.05). Additionally, The time of getting out of bed, fasting time, exhausting time, the retention time of the thoracic drainage tube, and the volume of chest drainage in the NIVATS group were significantly shorter than in the IVATS group (*P* < 0.05). Compared with the IVATS group, a hospital stay of the NIVATS group is significantly shorter, and shorter hospitalization cost and a lower incidence of POD (*P* < 0.05).

**Table 5:** The Recovery Indicators

	IVATS (*n* = 60)	NIVATS (*n* = 60)	*P*-value
Duration of awakening, (min)	14.05 ± 6.31	7.5 ± 4.16	< 0.0001
Extubation (LMA) time,(min)	16.43 ± 7.42	8.98 ± 5.64	< 0.0001
Chill	9 (15.00)	0 (0)	< 0.01
Agitation	6 (10.00)	0 (0)	0.03
Nausea/vomiting	6 (10.00)	3 (5.00)	0.5
Sore throat	18 (30.00)	6 (10.00)	< 0.01
Time to get out of bed, (h)	17.53 ± 4.07	15.18 ± 3.59	< 0.01
Fasting time, (h)	15.88 ± 3.45	14.02 ± 2.47	< 0.001
Exhausting time, (h)	23.15 ± 8.85	19.23 ± 8.43	0.01
Retention time of thoracic catheter, (h)	98.53 ± 22.47	89.58 ± 12.92	0.01
Chest drainage volume (ml)	885.68 ± 311.4	719.87 ± 295.71	< 0.01
hospital stay (d)	4.52 ± 1.10	4.12 ± 0.85	0.03
hospitalization cost (RMB)	75,219.3 ± 14,426.3	66,188.6 ± 14,361.1	< 0.001
POD	6 (10)	0 (0)	0.03
duration of antibiotic use (h)	87.7 ± 15.91	84.03 ± 10.25	0.34
VAS extubation_5min,_1	38 (63.33)	8 (13.33)	< 0.001
VAS extubation_15min,_1	35 (58.33)	11 (58.33)	< 0.001

Data are presented as the mean ± SD or number of patients (%)

*LAM* laryngeal mask, *POD* postoperative delirium, *VAS* visual analog scale, *SD* standard deviation; Pain grading: 1 = no pain, 2 = mild pain, 3 = moderate pain, 4 = severe pain

## Discussion

Previous research has suggested that NIVATS may potentially decrease the occurrence of PPCs [4, 24]. And there are no clinical studies demonstrating an increased incidence of PPCs in patients with NIVATS. The results of our study indicate that there was no statistically significant difference in the occurrence of PPCs between the two groups. Specifically, the incidence of PPCs in the NIVATS group was found to be comparable to that of the IVATS group, suggesting that NIVATS is not inferior to IVATS in terms of PPC incidence as the primary outcome.

The absence of a notable rise in PPCs in the NIVATS group can be attributed to the following reasons. First, pulmonary wedge resection and radical resection of the lung were only selected in our study, and the type of operation was relatively absolute and consistent. Second, all patients were operated by the identical senior anesthesiologist (more than 10 years) and the same group of surgeons in our study. Favorable anesthesia management and skilled surgical techniques, coupled with strict coordination, may effectively reduce PPCs. Third, the outcome could potentially be associated with the utilization of PVB in the NIVATS group. On the one hand, previous studies have shown that PVB can successfully reduce coughing pain. This analgesic effect is a benefit for sputum expulsion, lung expansion, and lung function recovery, and ultimately reduces PPCs incidence [25, 26]. On the other hand, the results of this study showed that the dose of perioperative anesthetic drugs used by patients in the NIVATS group was reduced, especially opioids. Numerous investigations [27, 28] have provided evidence that PVB and other nerve blocks can greatly decrease the need for intraoperative analgesics, and avoid respiratory depression, nausea and vomiting, and other related side effects, which in turn reduces PPCs and promotes rapid postoperative recovery.

In addition, the safety of NIVATS was a common concern. SPO_2_ and cough/body movement are the important safety indicators. There was no significant difference between the two groups in the incidence of SPO_2_ < 90% lasting more than 5 min and cough/body movement. PVB and homolateral vagal/phrenic nerve block for NIVATS could reduce the occurrence of coughing/body movement effectively [12, 29]. Besides, the higher ETCO_2_ and regurgitation/aspiration were also concerns in NIVATS, ETCO_2_ can return to the normal level 15 min after the extubation, which is consistent with the previous study [22, 30]. The change of ETCO_2_ will directly lead to the alteration of PaCO_2_, which causes the changes of PH, BE, and HCO_3_^−^. The results of this study showed that intraoperative PH and BE were significantly lower and PaCO_2_ and HCO_3_^−^ were significantly higher in the NIVATS group than in the VATS group, but HCO_3_^−^ and BE were within the normal range, and PaCO_2_ and PH were within the range of permissive hypercapnia (PHC). Related studies have found that PHC not only has a protective effect on the lungs, but also on other vital organs such as the brain and heart.

Furthermore, it is worth noting that these indicators were restored to their normal levels within 15 min following the surgical procedure, without any observed negative consequences such as malignant arrhythmia.

In our study, rSO_2_ in the NIVATS group was significantly higher than that in the IVATS group (T1-T6). The explanation could be that the elevated PaCO_2_ causes the expansion of cerebral blood vessels (CBV) and an increase of cerebral blood flow (CBF) [31, 32], eventually leading to an elevation in rSO2 [33]. Olesen et al. established a linear correlation between CBF and PaCO_2_ within the range of 25–65 mmHg. For every 1 mmHg increase in PaCO_2_, whole CBF increased by 1-2 ml·100 g^−1^ min^−1^, and CBV increased by approximately 1% [34]. Simultaneously, an increased PaCO_2_ exerts a notable constriction on the pulmonary vasculature [35], which might be likened to hypoxic pulmonary vasoconstriction. Consequently, this mechanism contributes to raised rSO_2_ and improved pulmonary oxygenation.

The recovery quality at NIVATS has been the focus of scrutiny. In this study, the awakening time and extubation time (LMA) were significantly shorter consistent with the higher of BIS (T5, T6) in the NIVATS group, which is consistent with the study by Guo Z et al. [36]. Possibly due to the absence of myorelaxant use during NIVATS, the residual effects of myorelaxation were avoided. Furthermore, the NIVATS group exhibited a marked reduction in the incidence of complications, including chills, agitation, sore pharynx, and pain scores, which played a crucial part in the patient's rapid recovery. Our results also confirmed that patients in the NIVATS group had significantly shorter times to get out of bed, fasting, expiration, chest drain retention, and volume of chest drain. Additionally, these patients had considerably shorter hospital stays and incurred far fewer hospital costs [37]. Previous studies [4, 22] have shown that the prognosis of elderly patients (median age 73 years) undergoing NIVATS is not inferior to that of IVATS, which was consistent with our research outcomes. Taking into account the above advantages, NIVATS was in line with the concept of accelerating rehabilitation surgery in thoracic surgery.

Our study showed that patients in the NIVATS group had relatively less dose of analgesic pumps and lower postoperative VAS scores. A previous study [38] reviewed the medical records of 384 patients who undergoing NIVATS also showed that analgesia tolerance of NIVATS was better, which was consistent with the results of our study. Administration of local anesthetic, nerve block, and fine operation may contribute to the results.

Currently, most studies of NIVATS focus on its feasibility and intraoperative safety. However, this study concentrated on the effects of NIVATS on patients' postoperative pulmonary complications, and explored the safety of this anaesthesia modality from the perspective of patients' prognosis, which provides a reference for clinical work. Our study also has limitations. Firstly, this is a single-center study with a small sample size. Secondly, the patient was restricted with ASA I ~ II and normal weight (BMI < 25 kg/m^2^), so the results may not represent the common patients. Thirdly, there is still insufficient long-term support for the benefits of NIVATS. We hope to conduct further research on the long-term survival and chronic pain of NIVATS.

## Conclusions

For patients with BMI < 25 kg/m^2^ and ASA I-II, in the NIVATS group, the incidence of PPCs was not inferior to that of the IVATS group. Furthermore, NIVATS had little effect on perioperative safety and substantially enhanced the quality of postoperative recovery. NIVATS may also become a new recommended method for thoracic surgery.

## Data Availability

All data generated or analysed during this study are included in this published article.
